# Cognitive Changes during Prolonged Stay at High Altitude and Its Correlation with C-Reactive Protein

**DOI:** 10.1371/journal.pone.0146290

**Published:** 2016-01-05

**Authors:** Sheng Li Hu, Wei Xiong, Zhi Qiang Dai, Heng Li Zhao, Hua Feng

**Affiliations:** 1 Department of Neurosurgery, Southwest Hospital, Third Military Medical University, Collaborative Innovation Center for Brain Science, Chong-Qing, 400038, China; 2 Department of Respiration, Southwest Hospital, Third Military Medical University, Chong-Qing, 400038, China; University of Miami School of Medicine, UNITED STATES

## Abstract

Hypersensitive C-reaction protein (hsCRP) may be a risk factor for cognitive impairment resulting from Alzheimer’s disease (AD), stroke, and vascular dementia. This study explored the correlation of peripheral blood hsCRP level with cognitive decline due to high altitude exposure. The study was conducted on 100 male military participants who had never been to high altitude. Cerebral oxygen saturation monitoring, event related potentials (P300, N200) detection, and neurocognitive assessment was performed and total hsCRP, interleukin-6 (IL-6), and homocysteine was estimated at 500m altitude, 3650m altitude, 3day, 1, and 3 month post arriving at the base camp (4400m), and 1 month after coming back to the 500m altitude. High altitude increased brain oxygen saturation, prolonged P300 and N200 latencies, injured cognitive functions, and raised plasma hsCRP levels. But they all recovered in varying degrees at 1 and 3 month post arriving at the base camp (4400m). P300 latencies and hsCRP levels were strongly correlated to cognitive performances. These results suggested that cognitive deterioration occurred during the acute period of exposure to high altitude and may recover probably owning to acclimatization after extended stay at high altitude. Plasma hsCRP is inversely correlated to neurological cognition and it may be a potential biomarker for the prediction of high altitude induced cognitive dysfunction.

## Introduction

High altitude is a stressful condition which is always related to a decreased oxygen supply to tissues [1]. Past studies have demonstrated that human brain takes up only about 2% of the body weight, but it accounts for about 20% of the oxygen consumption [2]. Thus, Brain in particular is highly vulnerable to such hypoxia exposure at high altitude. A wide range of evidences have shown hypobaric hypoxia at high altitude results in a myriad of neurophysiological and neurocognitive dysfunction including vision, memory, language, mood, learning, and processing speed [3–5]. Further studies found that long term stay at high altitude caused psychological and cognitive impairment in adults [6, 7] while not in adolescents [8]. Moreover, neuropsychological deficits may be persistent even after coming back to sea level from high altitude regions [9]. With the continued growth of people travelling to high altitude due to various reasons, the cognitive impairments resulted from hypoxia are of major public health importance and have economic consequences.

Growing evidences suggest that inflammation is a risk factor for neurodegenerative disorders such as Alzheimer’s disease (AD) and vascular dementia [10, 11]. Another study clearly indicated increasing levels of C-reactive protein (CRP) were associated with brain white matter integrity loss in corticosubcortical pathways [12]. Besides, recent reports have demonstrated a positive relationship between peripheral blood levels of CRP [13, 14] and neurocognitive decline. Another inflammation biomarker interleukin-6 (IL-6) also correlated inversely with cognitive performance in middle and old aged people [15, 16]. In addition, previous data show that homocysteine, a product of methylation reactions, may contribute to the pathophysiology of stroke [17], Schizophrenia [18], and the cognitive changes associated with normal aging [19, 20]. However, the association of hypersensitive CRP (hsCRP), IL-6, and homocysteine in humans with cognitive function during longitudinal exposure to high altitude is still unknown and remains to be explored.

Event related potentials (ERP) including P300 and N200, indexes of the sensory speed and cognitive performance, can reflect cognitive functions like cognitive processing and performance monitoring objectively. Therefore, the purpose of this study was to investigate the longitudinal cognitive test performance of people after prolonged stay at high altitude accompanied by ERP detection and cerebral oxygen saturation monitoring and to explore its correlation with peripheral blood hsCRP, IL-6, and homocysteine. A longitudinal study was performed on a relatively large cohort of 100 Chinese lowlander population who travelled to an altitude of 4300–4500 m (Tibet regions) from 300 m altitude (Si-Chuang province, China P.R.) and stayed there for about 3 months.

## Materials and Methods

The study was performed according to the Declaration of Helsinki and the experimental design and procedures for conducting the experiment were approved by the ethics committee on human investigation of Southwest Hospital, Third Military Medical University. Informed written consent was obtained from all the participants or their caretakers (2 subjects aged 17) who volunteered for the study. Before giving written and verbal consent to participate, each volunteer was informed of the possible risk and discomforts involved in the study. They all agreed to join the study by signing the informed consent documents, which has been approved by the ethics committee.

### Subjects

A total of 100 military participants comprising of male subjects with education of 9–16 yr and between age group of 17–34 yr enrolled voluntarily after being explained about the study purpose, protocol, and expected outcomes. They are lowlanders born and living at sea level below 500m and they are without any prior exposure to 4400 m altitude prior to their participation. They all have no documented neurological disorder or history of head injury with loss of consciousness and no previous history of drug abuse, stroke, epileptic seizures. The medical follow-up was performed during the expedition and physical examinations were used to determine health status.

### Expedition to Qinghai-Tibet Plateau and experimental protocol

**Fig 1:**
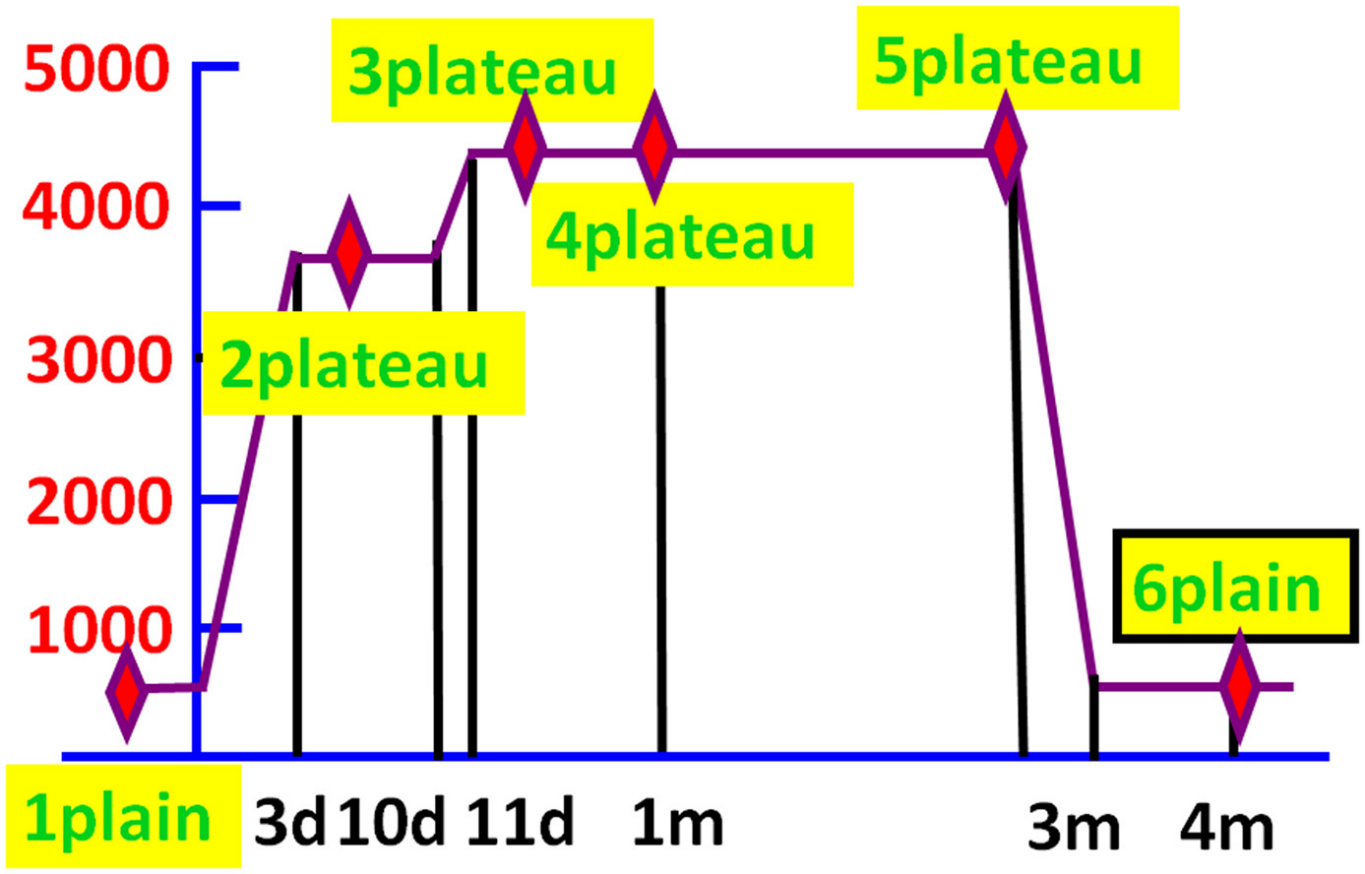
Climbing schedule of the expedition to Qinghai-Tibet Plateau. The six red rhombuses represented the six time points for investigations that were performed at 500m altitude (1 plain), 3650m altitude (2 plateau), 3day (3 plateau), 1 month (4 plateau), and 3 (5 plateau) month post arriving at the base camp (4400m), and about 1 month (6 plain) after coming back to the 500m altitude respectively.

During days 1–3, the volunteers left Si-Chuang province (500m) for Lhasa, Tibet (3650 m) by train and stayed there for 7 days. Then the subjects spent 1 day reaching the base camp (4400m) and lived in there for about 3 months. They came back to Si-Chuang province approximately 3 months later and completed the expedition. Total 6 examinations including heart rate (HR), pulse oxygen saturation (SPO_2_), brain oxygen saturation, auditory evoked event-related potentials (ERP, including P300 and N200), peripheral blood hypersensitive C-reactive protein (hsCRP), homocysteine, and interleukin-6 (IL-6) accompanied by neurological cognitive tests such as digit symbol substitution test (DSST) was administered at 500m altitude (1 plain-first point), 3650m altitude (2 plateau- second point), during the follow-ups at 3day (3 plateau-third point), 1 (4 plateau-fourth point), and 3 (5 plateau-fifth point) month post arriving at the base camp (4400m), and about 1 month (6 plain-sixth point) after coming back to the 500m altitude (Fig 1).

### Examinations of HR, SPO2, and brain oxygen saturation

One kind of oximeter (MD300C29, Chaosi corp. ltd, Peking, China) was used to non-invasively record HR and SPO_2_ at rest with probes attached to ring finger. Once the signal was steady, HR and SPO_2_ was produced by the apparatus.

Brain oxygen saturation monitor using non-invasive near-infrared spectroscopy technique (MNIR-P100, MingXi Corp. ltd, ChongQing, China) was applied to detect the brain frontal cortex oxygen saturation at rest with probes placed at the forehead. Left and right brain oxygen saturation was recorded once the signal was stable.

### Digit symbol substitution test (DSST)

DSST is a paper-and-pencil test derived from the Wechsler Adult Intelligence Scale-Revised. This examination measures response, attention, and processing speeds [21]. The test consists of nine digit-symbol pairs followed by a list of digits. Under each digit, the subject must write down the corresponding symbol as quickly as possible. In our study, the number of correct responses in 180s was recorded as the score. All subjects were seated in a conference room and took the test at the same time.

### Auditory evoked ERP (P300 and N200)

The test was performed using an auditory evoked potentials system (Keypoint 9033A07, Alpine Corp., Denmark) in an electrically shielded sound-proof room, with the volunteers lying comfortably, with eyes closed, in order to eliminate the artifacts caused by eye movement. Electrodes were positioned as follows: reference electrode situated at Cz, recording electrode linked to right and left earlobes. The impedance between electrodes was less than 5 KΩ. The click stimulus was presented through an earphone. The following parameters were used for the acquisition of P300 and N200: A random sequence of binaural tones (60 dB, 10 ms rise and fall, 50 ms plateau time) was presented, with a standard tone (1000 Hz) 70% of the time and a target tone (2000 Hz) 30% of the time. The latency of the largest positive potential occurring between 250 and 500 ms was designated the P300 component. The latency of the largest negative potential occurring between 175 and 250 ms was designated the N200 component.

### Biochemical tests

The blood samples for homocysteine and hsCRP test were taken into tubes containing dry heparin from the volunteers in aseptic conditions by venous puncture after an overnight fast and were immediately centrifuged at +4°C. Plasma was then separated and collected in another tube for freezing at -20°C, and stored at this temperature until assay. Serum for IL-6 test was extracted from blood samples by centrifugation and stored at -20°C for assay.

The homocysteine levels in plasma were determined by an assay kit (JiuQiang Corp. ltd, Peking, China) using biochemistry analyzer (AU5400, Olympus, Japan). The plasma hsCRP levels were determined by a kit (Orion Diagnostica Oy, Finland) according to the manufacturer’s instructions. Serum IL-6 was checked by an assay kit (Roche, Switzerland) with a fully automatic electrochemical luminescent immune analyzer (E170, Roche, Switzerland).

### Statistical analysis

All data were reported as means ± SD. Statistical analysis of the data was performed by repeated measure ANOVA using software SPSS 18.0. The correlation among the DSST performance, P300, and hsCRP was analyzed using Pearson method with software SPSS 18.0. A probability value of less than 0.05 was considered statistically significant.

## Results

### Changes of HR, SPO2, and brain oxygen saturation

**Fig 2:**
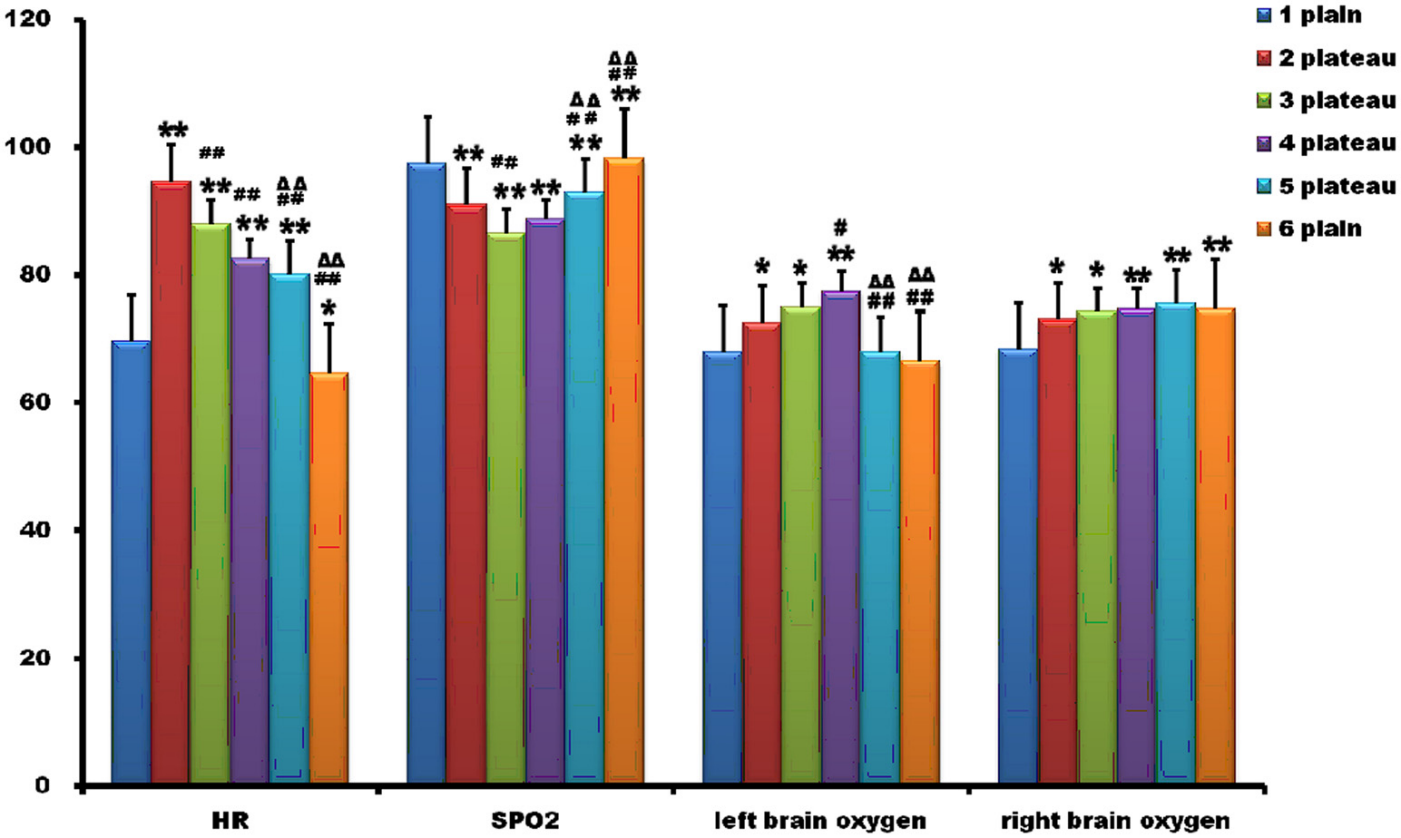
Changes of heart rate, pulse oxygen saturation, and brain oxygen saturation. The heart rate (HR) declined gradually after a rise during the stay at high altitude. Even at the sixth point, about 1 month after coming back to the 500m altitude, it continued to decrease. Pulse oxygen saturation (SPO_2_) displayed a downward state at high altitude, although it increased at 1 and 3 months post arriving at the base camp. The left and right brain oxygen saturation increased at the second, third, and fourth point, which was significantly higher than that at the first point. At the fifth and sixth point, left brain oxygen saturation nearly returned to normal, while the right brain oxygen was still at high level. *, p<0.05 compared with the first point; **, p<0.01 compared with the first point; #, p<0.05, compared with the second point; ##, p<0.01, compared with the second point; ΔΔ, p<0.01, compared with the third point.

The HR, SPO_***2***_, and brain oxygen saturation was depicted in Fig 2. The HR declined gradually after a rise during the stay at high altitude. At the second point (2 plateau), HR was significantly higher than that of the first point. Then, HR showed a downward trend. Even at the sixth point, around 1 month after coming back to the 500m altitude, it continued to decrease and differed statistically from that of the first point.

SPO_2_, an indicator of tissue oxygen, displayed a downward state at high altitude, although it increased at 1 and 3 month post arriving at the base camp (4 plateau and 5 plateau). SPO_2_ of all the four high altitude time points was statistically different from that of the first point (Fig 2).

We further examined brain oxygen saturation directly using non-invasive near -infrared spectroscopy technique. The left and right brain oxygen saturation increased at the second, third, and fourth point, which was significantly higher than that at the first point. At the fifth (5 plateau) and the sixth (6 plain) point, the left brain oxygen nearly returned to normal, while the right brain oxygen was still at high level (Fig 2).

### High altitude prolonged latencies of P300 and N200

**Fig 3:**
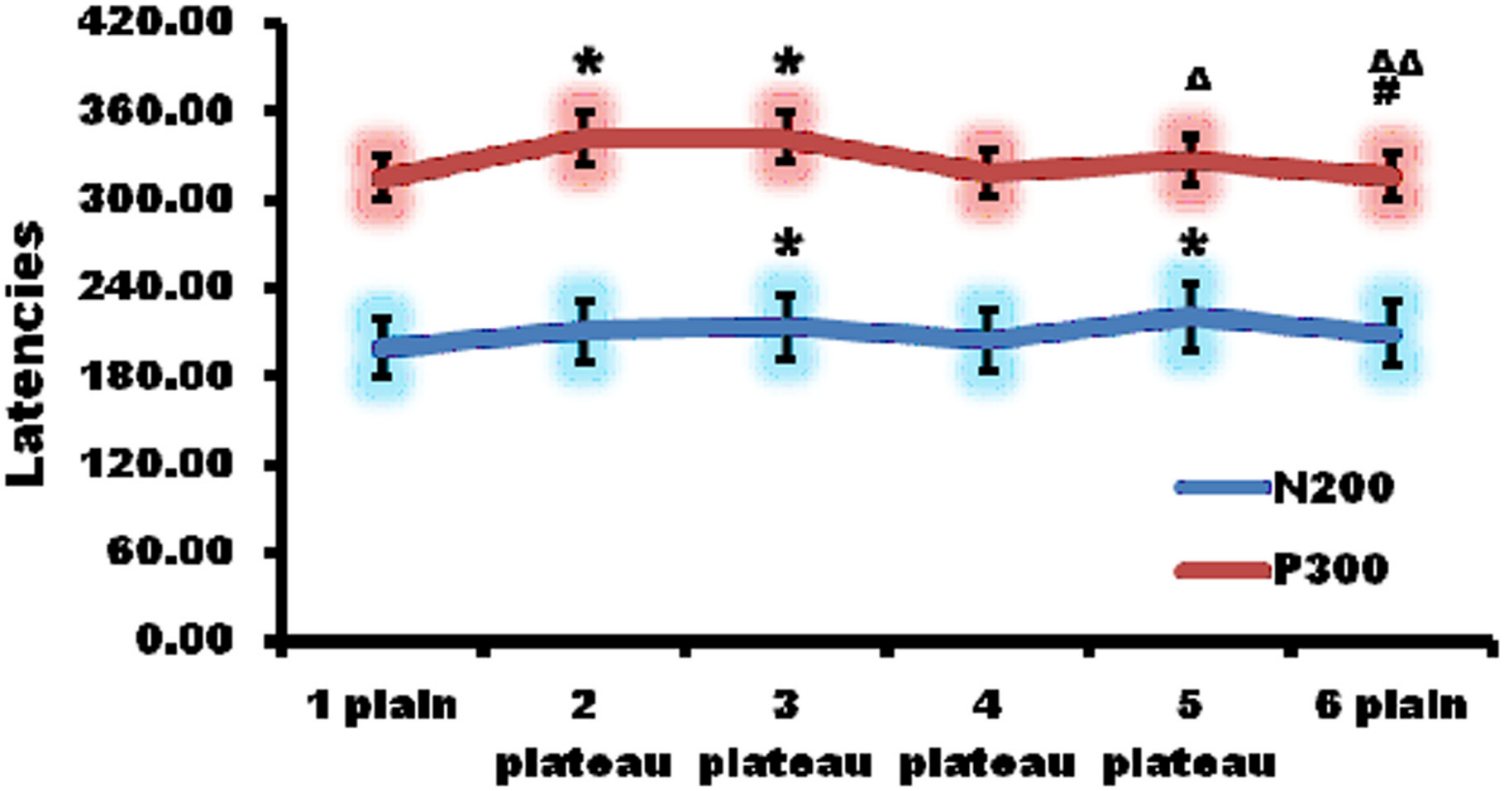
High altitude prolonged the latencies of auditory evoked event related potential (ERP). The latencies of P300 prolonged at the second and third point, which were significantly longer than that of the first point respectively. At the fourth point, the latency shortened. In addition, the latencies at the fifth and sixth point nearly returned to normal. N200 latency went up at the second point and increased much more at the third point than at the second. At the sixth point N200 latency returned to normal. *, p<0.05 compared with the first point; #, p<0.05, compared with the second point; Δ, p<0.05, compared with the third point; ΔΔ, p<0.01, compared with the third point.

Then P300 and N200 was quantitatively detected by auditory evoked ERP (Fig 3). The latencies of P300 prolonged at the second and third point, which were significantly longer than that of the first point respectively. Afterwards, the latencies shortened at the fourth, fifth, and sixth points, which showed no statistical significances compared to that of the first point.

N200 latency went up at the second point and increased much more at the third point than at the second. At the sixth point N200 latency returned to normal level. There was no statistical significance in N200 latency between the sixth and first point.

### High altitude increased plasma hsCRP levels

**Fig 4:**
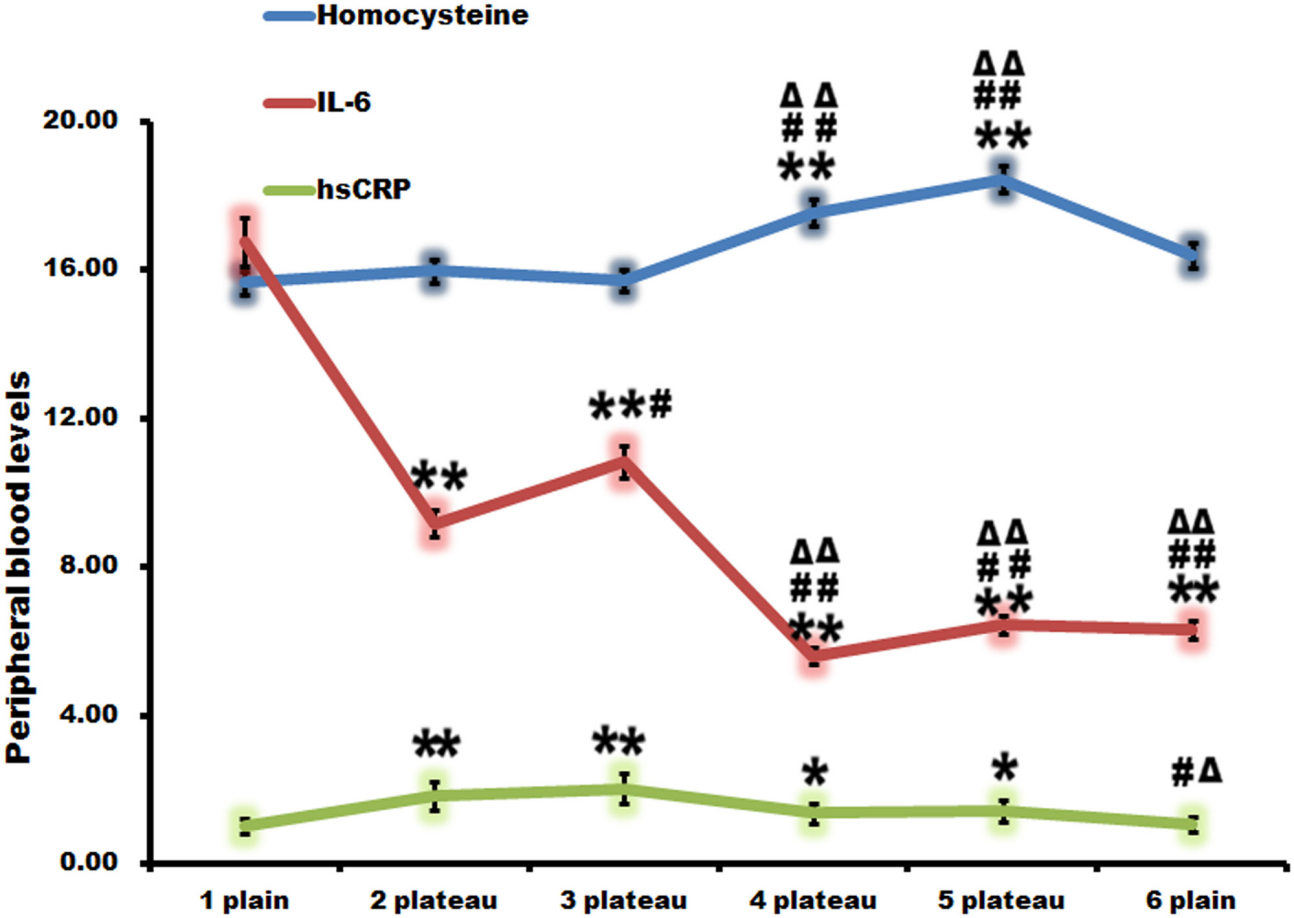
High altitude increased plasma hypersensitive C-reactive protein (hsCRP) levels. hsCRP augmented evidently at the second and third point. Then, it showed a downward tendency. At the sixth point it returned to normal. There was no obvious regularity in the alteration of homocysteine and IL-6. *, p<0.05 compared with the first point; **, p<0.01 compared with the first point; #, p<0.05, compared with the second point; ##, p<0.01, compared with the second point; Δ, p<0.05, compared with the third point; ΔΔ, p<0.01, compared with the third point.

Biochemical parameters such as hsCRP, homocysteine, and interleukin-6 (IL-6) were then investigated (Fig 4). hsCRP augmented evidently at the second and third point, which was much higher than that of the first point. Then, it showed a downward trend. At the sixth point, it returned to normal, which were statistically different from that at the second and third point. There was no statistical difference while compared with that of the first point.

The changes of homocysteine and IL-6 were also shown in Fig 4. They revealed no obvious alteration regularity.

### High altitude injured neurological cognitive functions

**Fig 5:**
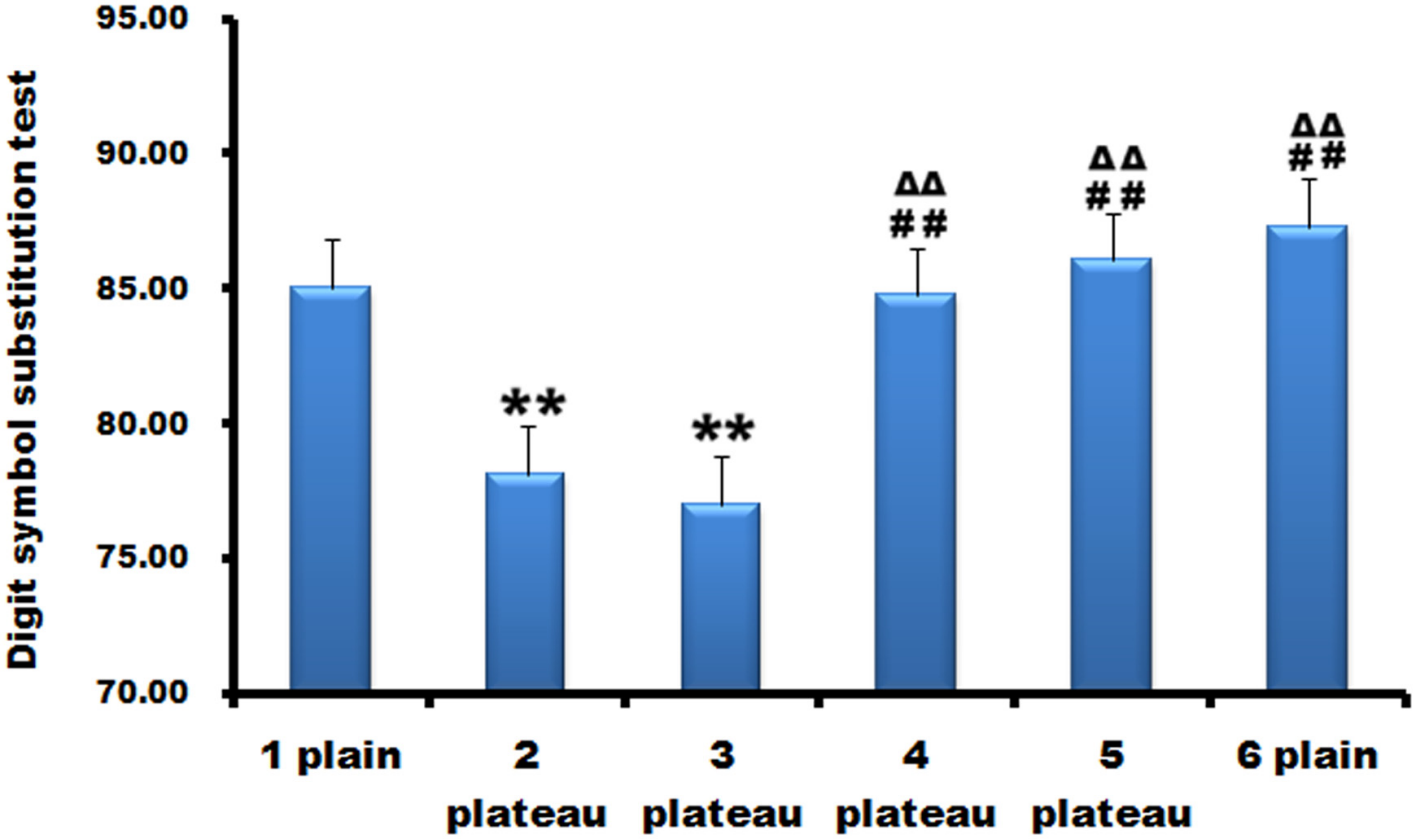
High altitude injured neurological cognitive functions. Digit symbol substitution test (DSST) score reduced at the second point and decreased much more at the third point than at the second. Scores at the fourth, fifth, and sixth point increased, which have no statistical differences compared to that of the first point. **, p<0.01 compared with the first point; ##, p<0.01, compared with the second point; ΔΔ, p<0.01, compared with the third point.

DSST was used for the evaluation of neurological cognitive functions (Fig 5). DSST score reduced at the second point and decreased much more at the third point than at the second. Scores at the fourth, fifth, and sixth point increased, which have no statistical differences compared to that of the first point.

### Association among biochemical parameters, ERP, and cognitive performance

**Fig 6:**
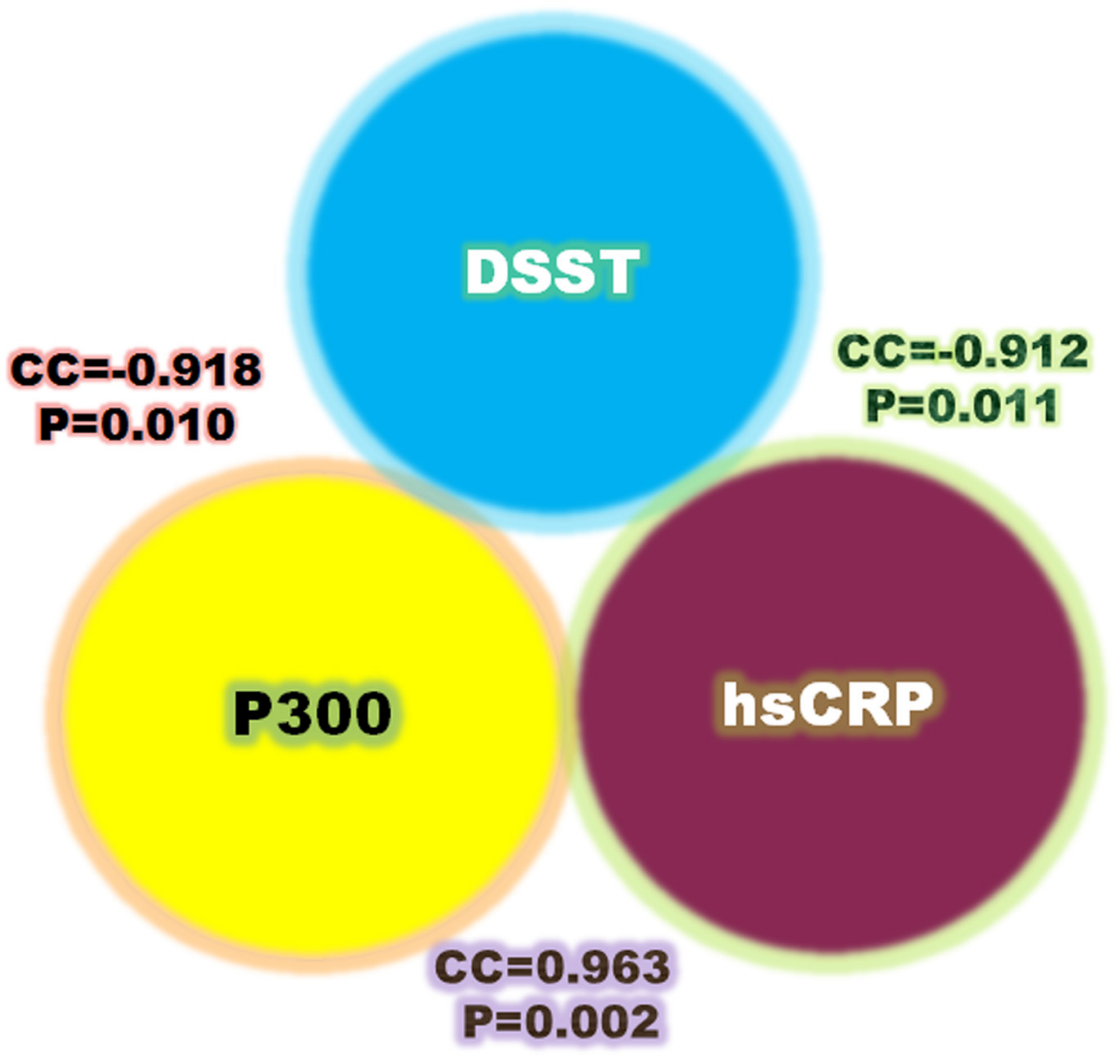
Associations among biochemical parameters, event related potential (ERP), and cognitive performance. Overall, P300 and hsCRP displayed the same changing tendency, which was opposite to the alteration of DSST score. A strong inverse correlation was observed between hsCRP and DSST score. P300 latency was also inversely correlated with DSST score. In addition, P300 latency showed a positive association with hsCRP.

Overall, P300 and hsCRP displayed the same changing tendency, which was opposite to the alteration of DSST score. Fig 6 illustrated Pearson’s correlation between DSST score and plasma hsCRP, a strong inverse correlation (correlation coefficient, CC = -0.912; p = 0.011) was observed between them. No significant correlation was found between DSST score and homocysteine or IL-6 (data not shown). P300 latency was also inversely correlated with DSST score (CC = -0.918; p = 0.010). In addition, P300 latency showed a positive association with hsCRP (CC = 0.963; p = 0.002).

## Discussion

The study provided evidence that high altitude increased brain oxygen saturation, prolonged P300 and N200 latencies, injured cognitive functions, and increased plasma hsCRP levels. But they all recovered in varying degrees at 1 and 3 month post arriving at the base camp (4400m). P300 latencies and hsCRP levels were strongly correlated to cognitive performances. These data clearly indicate that cognitive impairments occur during the acute and not the prolonged duration of exposure to altitude. Plasma hsCRP may be a potential biomarker for the prediction of high altitude induced cognitive dysfunction.

In agreement with other studies [22], resting heart rate was significantly higher upon arrival at high altitude than at 500m altitude. Then it declined gradually even after rise of altitude (4300-4500m). About 1 month post coming back to the 500m altitude, it continued to decrease, which was statistically different from that of the 500m altitude. Interestingly, most military volunteers developed bradycardia 1 month after departing from altitude. In contrast to the changes of pulse oxygen saturation, the left and right brain oxygen saturation increased at 3 day upon arrival at high altitude and 3 day and 1 month post arriving at the base camp (4400m) compared with baseline values. Three month after ascent to the altitude, left brain oxygen saturation nearly returned to normal, while the right brain oxygen was still at high level. However, past studies demonstrated the resting regional brain oxygen saturation in the frontal cortex decreased progressively following ascent to high altitudes [23, 24], which was inconsistent with the present results. The method monitoring brain oxygen saturation applied in these studies are the same near-infrared spectroscopy, which is a non-invasive technique based on the differential absorptive properties of oxyhaemoglobin and deoxyhaemoglobin for near infrared light (700–1100 nm) and has been used extensively in research despite its poor clinical use. The underlying reason for the difference is not well known and deserves further research. It may be related to the good physical fitness of the subjects in this study. In addition, we found an interesting phenomenon that the right brain oxygen was still at high level even 3 months after ascent to high altitude. The potential mechanism remains unclear which may be associated with different function of the left and right brain and needs to be elucidated in future study.

Then the cognitive potentials including P300 and N200 were quantitatively detected by auditory evoked ERP. ERP components including P300 and N200, indexes of the sensory speed and cognitive performance, enables direct examination of mental operations ranging from cognitive processing to performance monitoring [25]. Previous studies have found that acute exposure to a hypoxic environment through fast altitude gain or hypobaric hypoxia induces increases of ERP latency, particularly for the P300 [26, 27]. However, the time course of ERP alteration at high altitude is still unclear. Our study showed that P300 latency prolonged after ascent to high altitude and recovered after extended exposure. It returned to normal at 1 and 3 month post staying at altitude. N200 latency displayed a similar changing trend to P300. This suggests people with chronic exposure to high altitude hypoxia could produce acclimatization or adaptation that occurs after a relatively short period of time, which is in line with the past report by Singh et al [26].

The relationship between inflammation and cognitive function has been extensively studied in recent years. Although there are some negative results [28], most obtained studies suggest the positive associations. Kliper and colleagues [29] reported that higher levels of C-reactive protein were associated with worse performance in cognitive tests after stroke. Further research demonstrated high hsCRP may be a marker of memory and visuo-spatial impairment in the elderly [13]. The mechanisms that hsCRP induces cognitive impairment may involve cytotoxicity and structural brain changes from inflammation [30]. An obvious association between smaller brain volumes as measured via magnetic resonance imaging and higher levels of CRP was observed by Jefferson et al and Satizabal et al [31, 32]. The present study found plasma hsCRP increased evidently following ascent to high altitude. Then, it decreased and returned to normal level after 3 months’ exposure to high altitude. Furthermore, hsCRP levels demonstrated an inverse strong correlation with cognitive scores and a positive significant association with P300 latencies, which is the index of cognitive performance. These findings indicate that hsCRP may be the possible marker of cognitive decline associated with acute exposure to high altitude.

IL6 is another inflammation cytokine that is thought to be related to cognitive performance. Previous report revealed that patients with vascular dementia had elevated IL-6 values [33]. Additionally, Jordanova and colleagues reported an association between cognitive activity and IL-6 in an African-Caribbean populations aged 55–75 years old [34]. However, this study did not replicate the above results in people during rapid ascent to high altitude, although there was a major change in IL-6 levels. We did not find the rules underlying the changes especially its alteration tendency similar to the cognitive changes. There was no statistical significance in the correlation between IL-6 and DSST performance.

Past reports have shown that elevation of homocysteine is associated with age related neurological disorders and chronic stress models [35]. Moreover, a recent study provided evidences that increases in circulation homocysteine were positively correlated to cognitive damage and duration of stay at high altitude [36]. Contrary to these findings, the present results revealed no elevation of plasma homocysteine at the initial ascent to altitude, which increased after prolonged (1 month and 3month) duration. However, Similar to IL-6, the changing tendency of homocysteine was not related to the changes of cognitive results without statistical significance.

The present study, to the best of our knowledge, is the first report on relationships among hsCRP, P300, and cognitive impairment of lowlander natives following extended stay for 3 months at high altitude. The results showed there were mutual correlations among them. These findings could be of considerable clinical relevance for occupational health at high altitude and have major public health importance with the sustained rise of people going to high altitude for different purposes.

In conclusion, this study reveals the novel findings that cognitive dysfunction occurs during the acute period of exposure to high altitude and may recover probably due to acclimatization after prolonged stay. Plasma hsCRP is strongly correlated to neurological cognition and it may be a potential biomarker for cognitive decline resulted from high altitude. A shortage of this study is that the cerebral oxygen saturation we examined is contrary to previous studies. The underlying reason is unknown and further research is needed to be explored in future work.
